# Multilevel analysis of the role of women’s empowerment on use of contraceptive methods among married Cambodian women: evidence from demographic health surveys between 2005 and 2014

**DOI:** 10.1186/s12905-020-01141-z

**Published:** 2021-01-06

**Authors:** Owen Nkoka, Daphne Lee, Kun-Yang Chuang, Ying-Chih Chuang

**Affiliations:** 1 School of Public Health, Taipei Medical University, Taipei City, Taiwan 110; 2 Institute of Health and Wellbeing, University of Glasgow, Glasgow, UK

**Keywords:** Women’s empowerment, Contraceptive use, Multilevel analysis, Cambodia, DHS

## Abstract

**Background:**

The use of contraceptives is an essential public health concept that improves overall safe motherhood and infant health. Women empowerment has been reported to influence health behaviors in women. With recent efforts to increase access to contraceptive methods, uptake of the same remains a challenge in Cambodia. There are limited studies that have examined the role of women’s empowerment at both individual- and community- level on contraceptive use in Cambodia. This study examined the individual- and community-level factors associated with contraceptive use among Cambodian married women between 2005 and 2014.

**Methods:**

Data from 2005, 2010, and 2014 Cambodia Demographic and Health Surveys were used to analyze 2211; 10,505; and 10,849 women, respectively. Multilevel binary and multinomial logistic regression models were applied to assess the association between individual- and community- level factors, and the use of contraceptive methods.

**Results:**

The prevalence of using modern contraceptive methods increased over time (i.e., 29.0, 38.1, and 42.3% in 2005, 2010, and 2014, respectively). At the individual level, women who attained secondary and higher education were more likely to use any contraceptives [adjusted odds ratio (aOR) = 1.43, 95% confidence interval (CI) = 1.22–1.68, and aOR = 1.23, 95% CI = 1.05–1.44 in 2010 and 2014, respectively] compared with those with no formal education. Similarly, having a high workforce participation level was significantly associated with increased likelihood of using any contraceptive methods [aOR = 1.12, 95% CI = 1.00–1.26, aOR = 1.44, 95% CI = 1.29–1.60 and in 2010 and 2014, respectively]. Other factors such as age at first marriage, residence, and having a health insurance were associated with contraceptive use. The proportional change in variance showed that about 14.3% of total variations in the odds of contraceptive use across the communities were explained by both individual- and community-level factors. Moreover, the intraclass correlation showed that about 5.2% of the total variation remained unexplained even after adjustments.

**Conclusion:**

Both individual- and community- level factors influenced contraceptive use in Cambodia. When designing programs to improve contraceptive use, contextual influences should be taken into account for the effectiveness of the programs.

## Background

Contraceptive use is an important public health issue that promotes safe motherhood and infant health [1–3]. Contraceptives may be used for appropriate child spacing which has been shown to reduce the likelihood of preterm births [4, 5]. Additionally, contraceptives may be used for limiting child bearing especially to avoid high risk pregnancies thereby, helping to reduce maternal and infant mortality [6, 7]. A multi-country study conducted in 2010 revealed that contraceptive use reduced maternal mortality globally by 44.0% [6]. Since 1987, a global initiative known as “Safe Motherhood” has been championed to reduce maternal mortality [8–10]. This initiative encompasses a wide range of issues relating to maternal health including family planning (FP) – one of the four pillars of safe motherhood [11].

An important component of FP is the use of contraceptives. FP is not only a significant health issue but also affects a wide range of determinants essential for the attainment of sustainable development goals [12]. Therefore, more emphasis has been placed in making modern FP methods accessible to the population to ensure good FP practices [13, 14]. However, the use of contraceptives has been shown to be influenced by a wide range of social, cultural, and religious factors [15–17]. One dimension that has been shown to influence contraceptive use is  women empowerment. For instance, a study in lower middle income countries reported an association between a woman’s decision making power within a household and the use of contraceptives [18]. Women with household decision-making power are more likely to have control of their bodies and fertility [19]. Further, attainment of high educational levels bequeaths women with knowledge and skills that promote their well-being [20]. Moreover, women’s involvement in workforce improves their economic independence which may ultimately help them have better access to healthcare services including contraceptives. Thus, education, workforce and decision making power have been widely promoted as important measurements for women empowerment [21, 22].

Cambodia experiences high rates of fertility and maternal mortality [23]. Despite several FP programs aimed to make contraceptives accessible, uptake of contraceptives has been a challenge [24]. A study was conducted in Cambodia to understand influence of social networks and contraceptive use [25]. Additionally, a recent Cambodian study revealed that women empowerment within the household was critical for the use of contraceptives [26]. To date, there is no study in Cambodia that examined the role of individual- and community- level factors in influencing contraceptive use. Further, few studies have examined empowerment at both individual- and community- level and how this influences contraceptive use [11, 27]. Community factors have been shown to influence health behaviors and access to services [28, 29]. It is, therefore, important to understand the influences of community characteristics on contraceptive use as findings from this research may help design effective FP programs that take into account the contextual factors.

Therefore, this study examined the influence of individual- and community- level factors, with an emphasis on women’s empowerment measures, on contraceptive use among married Cambodian women between 2005 and 2014.

## Methods

### Study design and data source

This cross-sectional study used data from the 2005, 2010, and 2014 Cambodia demographic and health surveys (CDHS). The surveys adopted a stratified two-stage cluster sampling  method to select households for the survey. The first stage involved the selection of enumeration areas (EAs) and household listing within the selected EAs. In the second stage, households were selected through equal probability sampling criterion. In 2005, 557 EAs were selected while 677 EAs were selected for both 2010 and 2014 surveys. Details of the sampling strategy of the 2005, 2010, AND 2014 CDHS have been published elsewhere [30–32]. A community was defined as the primary sampling unit (i.e., enumeration areas) of the CDHS. Face to face interviews were conducted to all women of the reproductive age (15–49 years) in the sampled households. Data analyzed in this study were from the individual (women’s) questionnaire of the CDHS which have been published elsewhere [20–32]. In 2005, of the 17,256 eligible women, 16,823 were successfully interviewed representing a 98% response rate. The subsequent surveys also yielded a 98% response rate (i.e., 18,754 of 19,237 in 2010 and 17,578 of 18,012 in 2014). The current analysis was restricted to women who were currently married/in union or living with a man (i.e., *n* = 2211 in 2005, *n* = 10,505 in 2010, and n = 10,849 in 2014).

### Measures

#### Outcome

The dependent variable in this study was contraceptive use defined as the use of either traditional or modern contraceptive methods. Modern methods of contraception included pills, female and male sterilization, intrauterine device, injectable, implants, male and female condom, and the diaphragm [33–35]. On the other hand, traditional methods of contraception included withdrawal, periodic abstinence, and folk methods [33–35]. Contraceptive use was categorized as a two-level (Any method and no method), and three-level (modern method, traditional method, and no method) variables.

#### Independent variables – individual-level factors

Three variables at individual-level were considered to measure women’s empowerment status. First, educational level was categorized as “no formal”, “primary”, and “secondary and tertiary”. Second, women’s household decision-making power was measured based on responses to individual questions regarding who has the final say in the family on the respondent’s health care, large household purchases, and visits to family or relatives. Response options included (a) respondent alone, (b) respondent and husband/partner, (c) respondent and other person, (d) husband/partner alone, (e) someone else, and (f) other. For each question, a value of 1 was assigned for responses of (a), (b), or (c) to designate high decision-making power, and 0 for (d), (e), or (f) to designate low power. A composite score was created for responses to the three dimensions of decision making power (i.e., health care, large household purchases, and visits to family/relatives) that ranged from 0 to 3. Participants were categorized as having low (a score of 0 to 1), middle (score of 2), and high (score of 3) household decision making power. Third, workforce participation consisted of current occupational status (yes or no), work consistency (respondent working throughout the year was coded as “1”, those working seasonally or just occasionally were coded as “0”), and payment type (respondent who reported to receive cash was coded as “1” while those paid in-kind ora combination of the two or not paid for their work were coded as “0”). A score was generated and grouped into three levels; low “0 to 1”, middle “score of 2”, and high “score of 3”.

Additionally, following a literature review [18, 36, 37], a wide range of individual-level factors were such as age (15–24, 25–34, or 35–49 years), religion (Buddhist, Muslim, or other), place of residence (urban or rural), region (plains, Tonle Sap, plateau / mountain, or Phnom Penh), age at first marriage (≤16, 17–20, or ≥ 21 years), total children ever born (0, 1–2, or ≥ 3), distance to health facility (big problem or not), health insurance coverage (no or yes), and husband/partner educational attainment (no education, primary, or secondary and higher). The CDHS assessed wealth index as a composite score measured from household assets such as televisions, bicycles [30–32]. The scores were grouped into quintiles from poorest to richest. For purposes of this research, wealth was grouped into 3-levels as poor (lower 40%), middle (middle 20%), and rich (upper 40%). Media exposure was measured by access to newspapers, radio, and television. Individuals reporting to have read newspaper, or watched television, or listened to radio at least once a week were categorized as having media exposure, otherwise, no.

#### Independent variables – community-level factors

The main independent variables at individual level were aggregated to form variables at community level. Community education, community workforce participation, community women’s decision making power, and community wealth were calculated as proportions of women with any education, with workforce participation, having power to make decisions, and from rich households, respectively. The continuous variables were then grouped into tertiles as low, middle, and high for easy interpretation of the results.

#### Data analysis

Weighted frequencies and percentages were reported for the selected factors. The statistical software Stata was used for analysis of the datasets. The “*svy*” command was used to account for the sampling weights and clustering effects of the Demographic and Health Survey (DHS). The weights were calculated according to DHS guidelines [38]. Multilevel binary and multinomial logistic regression models were used to examine the association of individual- and community-level factors and contraceptive use. The binary multilevel logistic model was assessed using the “*xtmelogit”* command in Stata. The multinomial logistic model was estimated using generalized structural equation modeling (GSEM) using the “*gsem”* command. Four models were fitted in each survey year. Model 1 included outcome variable only, model 2 included the outcome and individual-level variables, model 3 included the outcome and community-level variables, and model 4 included the outcome and both individual and community-level factors. Fixed effects were reported as adjusted odds ratio (aOR) with 95% confidence intervals (CIs). Random effects were reported as area variance, intraclass correlation coefficient (ICC), proportional change in variance (PCV), and median odds ratio (MOR). Model goodness of fit was checked by Akaike information criterion (AIC) with lower AIC suggesting a better fit. All analyses were performed using Stata version 15 (Stata Corp, College Station, TX, USA) and significance level was set at *p* < 0.05.

#### Ethical considerations

The survey protocol was reviewed and approved by the Cambodia National Ethical Committee for Health Research. Informed consent for the surveys was obtained from each respondent at the start of each interview. Clearance to analyze the data was provided by the Demographic Health Survey (DHS) program. The data is publicly available and may be requested from the DHS program through https://dhsprogram.com/data/available-datasets.cfm.

## Results

### Descriptive results

**Fig. 1:**
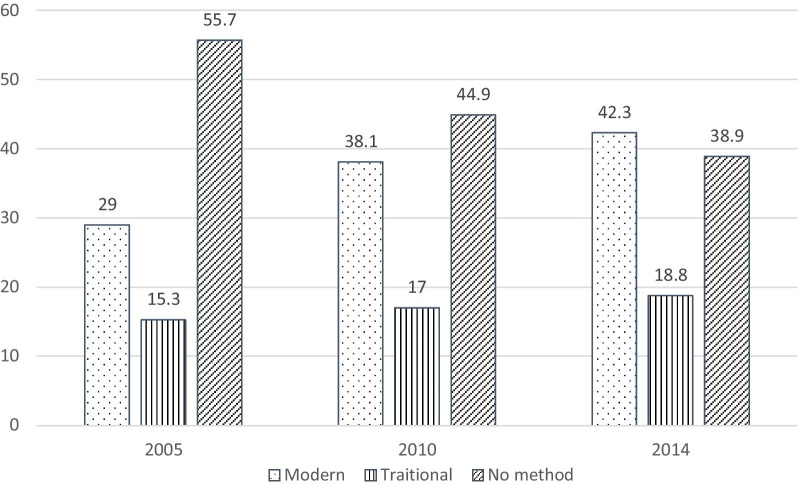
Prevalence of contraceptive use according to type of contraception method

The prevalence of contraceptive use is displayed in Fig. 1. Approximately 29.0% women reported using modern contraceptive methods in 2005 while 38.1% in 2010 and 42.3% in 2014 used modern contraceptive methods.

Table 1 lists the descriptive characteristics of 2211 women in 2005 (nested in 556 communities), 10,505 women in 2010 (nested in 611 communities), and 10,849 women in 2014 (nested in 611 communities). Among others, in all the three surveys, a majority of the women had primary education, high decision making power within their households, aged ≥35 years, Buddhist, from rural areas, and had media exposure. Workforce participation increased over time with 25.0, 29.6, and 39.0% of the women having high workforce participation in 2005, 2010, and 2014, respectively.

**Table 1:** Descriptive of individual- and community level characteristics of study sample

Variables	2005 (n = 2211)	2010 (*n* = 10,505)	2014 (n = 10,849)
	n (%)	n (%)	n (%)
Educational level			
No formal education	498 (22.5)	1995 (19.0)	1662 (15.3)
Primary	1323 (59.8)	5907 (56.2)	5886 (54.3)
Secondary+	390 (17.7)	2603 (24.8)	3301 (30.4)
Decision-making			
Low	92 (4.2)	400 (3.8)	382 (3.5)
Middle	409 (18.5)	1056 (10.1)	1052 (9.7)
High	1710 (77.3)	9049 (86.1)	9415 (86.8)
Workforce participation			
Low	890 (40.2)	4424 (42.1)	3393 (31.3)
Medium	770 (34.8)	2972 (28.3)	3223 (29.7)
High	551 (25.0)	3109 (29.6)	4233 (39.0)
Age (years)			
15–24	368 (16.6)	1615 (15.4)	1795 (16.5)
25–34	732 (33.1)	3898 (37.1)	4426 (40.8)
≥ 35	1111 (50.2)	4992 (47.5)	4628 (42.7)
Religion			
Buddhist	2142 (96.9)	10,218 (97.3)	10,413 (96.0)
Muslim	39 (1.8)	146 (1.4)	207 (1.9)
Other	30 (1.3)	141 (1.3)	229 (2.1)
Residence			
Urban	353 (16.0)	1893 (18.0)	1627 (15.0)
Rural	1858 (84.0)	8613 (82.0)	9222 (85.0)
Region			
Plains	898 (40.6)	4345 (41.4)	4093 (37.7)
Tonle Sap	834 (37.7)	3695 (35.2)	4003 (36.9)
Plateau / Mountain	268 (12.1)	1448 (13.8)	1788 (16.5)
Phnom Penh	211 (9.6)	1017 (6.6)	965 (8.9)
Age at first marriage			
≤ 16	423 (19.2)	1928 (18.3)	2022 (18.6)
17–20	1078 (48.7)	5070 (48.3)	4956 (45.7)
≥ 21	710 (32.1)	3507 (33.4)	3871 (35.7)
Total children ever born			
0	82 (3.7)	444 (4.2)	600 (5.5)
1–2	788 (35.6)	4450 (42.4)	5183 (47.8)
≥ 3	1341 (60.7)	5611 (53.4)	5066 (46.7)
Wealth			
Poor	886 (40.0)	4136 (39.4)	4293 (39.5)
Middle	417 (18.9)	2059 (19.6)	2173 (20.1)
Rich	908 (41.1)	4310 (41.0)	4383 (40.4)
Media exposure			
No	573 (25.9)	3715 (35.4)	3721 (34.3)
Yes	1638 (74.1)	6790 (64.6)	7128 (65.7)
Perceived distance to HF			
Big problem	897 (40.6)	6842 (65.1)	3956 (36.5)
No problem	1314 (59.4)	3663 (34.9)	6893 (63.5)
Health insurance			
No	–	9409 (89.6)	9172 (84.5)
Yes	–	1096 (10.4)	1677 (15.5)
Partner education			
No formal education	294 (13.3)	1265 (12.0)	1072 (9.9)
Primary	1186 (53.7)	4845 (46.1)	4995 (46.0)
Secondary and higher	731 (33.0)	4395 (41.9)	4782 (44.1)
Contraceptive use			
No	1232 (55.7)	4716 (44.9)	4221 (38.9)
Traditional	339 (15.4)	1790 (17.0)	2043 (18.8)
Modern	640 (28.9)	3999 (38.1)	4585 (42.3)
*Community-level factors*			
Community wealth			
Low	658 (29.7)	3706 (35.3)	3778 (34.8)
Middle	841 (38.1)	3999 (38.1)	4426 (40.8)
High	712 (32.2)	2800 (26.6)	2645 (24.4)
Community education			
Low	675 (30.5)	2810 (26.8)	3481 (32.1)
Middle	749 (33.9)	3668 (34.9)	3872 (35.7)
High	787 (35.6)	4027 (38.3)	3496 (32.2)
Community decision-making			
Low	569 (25.7)	3643 (34.7)	3430 (31.6)
Middle	723 (32.7)	3571 (34.0)	3576 (33.0)
High	919 (41.6)	3291 (31.3)	3843 (35.4)
Community workforce participation			
Low	788 (35.6)	3388 (32.3)	3941 (36.3)
Middle	751 (34.0)	3795 (36.1)	3897 (35.9)
High	672 (30.4)	3322 (31.6)	3011 (27.8)

*HF* Health facility

#### Empowerment factors associated with use of any contraceptive method

Table 2 displays the adjusted effects of a wide range of individual- and community level factors on contraceptive use (i.e., any method vs no method). Results from model 4 have been emphasized as the model displayed better fit.

**Table 2:** Multilevel logistic analysis of factors associated with contraceptive use in Cambodia

Variables	2005	2010	2014
	Any method vs. no method aOR (95% CI)	Any method vs. no method aOR (95% CI)	Any method vs. no method aOR (95% CI)
**Individual-level factors**			
Educational level			
No formal education	1.00	1.00	1.00
Primary	1.12 (0.85–1.47)	**1.14 (1.01–1.29)**	**1.17 (1.02–1.34)**
Secondary+	1.34 (0.91–1.96)	**1.43 (1.22–1.68)**	**1.23 (1.05–1.44)**
Decision-making			
Low	1.00	1.00	1.00
Middle	1.08 (0.65–1.80)	0.94 (0.72–1.23)	1.17 (0.89–1.54)
High	1.09 (0.66–1.77)	0.99 (0.77–1.25)	1.21 (0.94–1.54)
Workforce participation			
Low	1.00	1.00	1.00
Medium	1.27 (0.99–1.62)	**1.14 (1.01–1.28)**	**1.27 (1.13–1.43)**
High	1.28 (0.97–1.69)	**1.12 (1.00–1.26)**	**1.44 (1.29–1.60)**
Age (years)			
15–24	1.00	1.00	1.00
25–34	1.21 (0.88–1.68)	**1.36 (1.18–1.57)**	**1.25 (1.08–1.43)**
≥ 35	0.79 (0.54–1.14)	**0.71 (0.61–0.84)**	**0.57 (0.49–0.67)**
Religion			
Buddhist	1.00	1.00	1.00
Muslim	0.88 (0.41–1.91)	0.93 (0.64–1.37)	0.89 (0.63–1.26)
Other	0.74 (0.39–1.41)	0.78 (0.57–1.08)	1.15 (0.87–1.53)
Residence			
Urban	1.00	1.00	1.00
Rural	0.89 (0.67–1.18)	0.97 (0.80–1.16)	**1.25 (1.05–1.49)**
Region			
Plains	1.00	1.00	1.00
Tonle Sap	**1.49 (1.13–1.96)**	1.04 (0.89–1.23)	1.11 (0.96–1.27)
Plateau / Mountain	**1.69 (1.23–2.32)**	0.99 (0.83–1.18)	1.05 (0.90–1.23)
Phnom Penh	1.29 (0.76–2.18)	0.92 (0.68–1.25)	0.80 (0.60–1.05)
Age at first marriage			
≤ 16	1.00	1.00	1.00
17–20	0.93 (0.72–1.20)	0.91 (0.81–1.02)	**0.87 (0.77–0.97)**
≥ 21	**0.70 (0.52–0.94)**	**0.75 (0.66–0.86)**	**0.66 (0.59–0.75)**
Total children ever born			
0	1.00	1.00	1.00
1–2	**6.40 (2.84–14.43)**	**13.19 (8.70–19.99)**	**13.58 (9.57–19.27)**
≥ 3	**6.89 (3.00–15.81)**	**14.98 (9.79–22.92)**	**16.94 (11.80–24.32)**
Wealth			
Poor	1.00	1.00	1.00
Middle	1.23 (0.93–1.62)	1.09 (0.96–1.24)	0.96 (0.84–1.09)
Rich	1.36 (0.99–1.87)	0.87 (0.75–1.01)	0.89 (0.77–1.03)
Media exposure			
No	1.00	1.00	1.00
Yes	1.14 (0.90–1.46)	1.06 (0.96–1.17)	0.98 (0.88–1.08)
Perceived distance to HF			
Big problem	1.00	1.00	1.00
No problem	0.98 (0.79–1.21)	1.03 (0.93–1.13)	1.03 (0.92–1.11)
Health insurance			
No	–	1.00	1.00
Yes	–	0.90 (0.79–1.03)	**1.25 (1.10–1.41)**
Partner education			
No formal education	1.00	1.00	1.00
Primary	1.22 (0.90–1.66)	**1.16 (1.00–1.34)**	0.99 (0.85–1.37)
Secondary and higher	1.19 (0.84–1.71)	1.15 (0.98–1.36)	1.04 (0.88–1.23)
**Community-level factors**			
Community wealth			
Low	1.00	1.00	1.00
Middle	1.05 (0.79–1.39)	0.92 (0.78–1.09)	1.02 (0.88–1.18)
High	0.92 (0.62–1.38)	0.87 (0.68–1.10)	1.10 (0.87–1.39)
Community education			
Low	1.00	1.00	1.00
Middle	1.15 (0.87–1.52)	0.92 (0.78–1.08)	**0.84 (0.72–0.97)**
High	0.97 (0.72–1.34)	**0.83 (0.69–0.99)**	**0.78 (0.66–0.91)**
Community decision-making			
Low	1.00	1.00	1.00
Middle	1.03 (0.78–1.36)	1.05 (0.90–1.23)	1.01 (0.88–1.17)
High	1.09 (0.82–1.45)	1.04 (0.88–1.23)	0.96 (0.83–1.11)
Community workforce participation			
Low	1.00	1.00	1.00
Middle	**1.38 (1.06–1.81)**	1.08 (0.92–1.27)	0.95 (0.82–1.09)
High	1.32 (0.99–1.76)^╕^	1.12 (0.94–1.23)	0.96 (0.74–1.04)
**Measures of variation**			
Area variance (95% CI)	**0.23 (0.10–0.57)**	**0.28 (0.23–0.36)**	**0.18 (0.13–0.24)**
ICC (%)	6.53	8.00	5.22
PCV (%)	23.33	3.45	14.29
MOR	1.58	1.66	1.50
**Model fit statistic**			
AIC	2770.24	13,127.01	13,487.78

^╕^borderline *p-*value, bold means *p*-value < 0.05

*aOR* Adjusted odds ratio*, CI* Confidence internal, *ICC* Intraclass correlation coefficient, *MOR* Median odds ratio, *PVC* Proportional change in variance, *AIC* Akaike information criterion

While educational level, women’s decision making power, and workforce participation were not associated with use of any contraceptive method in 2005, significant associations were observed in 2010, and 2014. Specifically, in 2010, women with primary [aOR: 1.14, 95% CI: 1.01–1.29] and secondary and tertiary [aOR: 1.43, 95% CI: 1.22–1.68] were more likely to use any contraceptive method compared with those having no formal education. Similarly, in 2014, compared with women with no formal education, increased odds were observed among those with primary [aOR: 1.17, 95% CI: 1.02–1.34] and secondary and tertiary [aOR: 1.23, 95% CI: 1.05–1.44].

The results in 2010 further revealed that women with middle workforce [aOR: 1.14, 95% CI: 1.01–1.28] and high [aOR: 1.12, 95% CI: 1.00–1.26] participation levels were more likely to use any contraceptive methods compared with those having low workforce participation. In 2014, similar associations were noted with increased odds observed among women with middle [aOR: 1.27, 95% CI: 1.13–1.43] and high [aOR: 1.44, 95% CI: 1.29–1.60] workforce participation levels.

At community level, in 2010, women from communities with high a high percentage of educated women were less likely [aOR: 0.83, 95% CI: 0.69–0.99] to use any contraceptive method compared with those from communities with a low percentage of educated women. Similarly, in 2014, women from communities with a middle [aOR: 0.84, 95% CI: 0.72–0.97] and a high [aOR: 0.78, 95% CI: 0.66–0.91] percentage of educated women were less likely to use any contraceptive methods. Additionally, it was observed in 2005 that women from communities with a middle percentage of women in workforce were more likely [aOR: 1.38, 95% CI: 1.06–1.81] to use any contraceptive method compared with those from communities with a low percentage of women in workforce.

#### Empowerment factors associated with use of specific contraceptive methods

Results from the multinomial logistic regression analyses are listed in Table 3. Results from model 4 are presented because the model had a better goodness of fit (i.e., lower AIC). Educational level was associated with increased odds of using both modern and traditional methods (with no method as base category) in 2010 and 2014. Having primary education was positively associated [aOR: 1.52, 95% CI: 1.01–2.75] with using traditional contraceptive methods in 2005. In 2010, the odds of using traditional methods were higher among those with primary [aOR: 1.41, 95% CI: 1.15–1.91], and secondary and tertiary education [aOR: 1.99, 95% CI: 1.57–2.05] compared with those having no formal education. Additionally, the odds of using modern methods were high among those with primary [aOR: 1.21, 95% CI: 1.06–1.38] and secondary and tertiary [aOR: 1.73, 95% CI: 1.46–2.05] education. Similarly, having secondary and tertiary education was positively associated with use of traditional method [aOR: 1.46, 95% CI: 1.15–1.85] compared with those having no formal education in 2014. Further, having primary [aOR: 1.23, 95% CI: 1.06–1.42] and secondary and tertiary [aOR: 1.38, 95% CI: 1.16–1.65] education was associated with increased likelihood of using modern contraceptive methods.

**Table 3:** Multilevel multinomial logistic regression analysis of factors associated with contraceptive use in Cambodia based om 2005, 2010, and 2014 (Final Models only)

Variables	2005		2010		2014	
	Traditional vs no method RRR (95% CI)	Modern vs no method RRR (95% CI)	Traditional vs no method RRR (95% CI)	Modern vs no method RRR (95% CI)	Traditional vs no method RRR (95% CI)	Modern vs no method RRR (95% CI)
**Individual-level factors**						
Educational level						
No formal education	1.00	1.00	1.00	1.00	1.00	1.00
Primary	**1.52 (1.01–2.75)**	1.20 (0.90–1.59)	**1.41 (1.15–1.91)**	**1.21 (1.06–1.38)**	1.22 (0.99–1.50)	**1.23 (1.06–1.42)**
Secondary+	1.54 (0.90–1.99)	1.45 (0.97–2.18)	**1.99 (1.57–2.52)**	**1.73 (1.46–2.05)**	**1.46 (1.15–1.85)**	**1.38 (1.16–1.65)**
Decision-making						
Low	1.00	1.00	1.00	1.00	1.00	1.00
Middle	1.32 (0.58–1.17)	1.11 (0.66–1.88)	1.36 (0.91–2.04)	1.01 (0.76–1.34)	1.22 (0.85–1.75)	1.26 (0.94–1.70)
High	1.26 (0.57–2.78)	1.09 (0.65–1.81)	**1.71 (1.18–2.47)**	1.13 (0.88–1.46)	1.05 (0.75–1.45)	1.23 (0.94–1.61)
Workforce participation						
Low	1.00	1.00	1.00	1.00	1.00	1.19 (0.99–1.44)
Medium	0.81 (0.56–1.17)	1.23 (0.95–1.59)	**1.48 (1.25–1.76)**	**1.24 (1.10–1.41)**	**1.53 (1.29–1.82)**	**1.45 (1.27–1.65)**
High	1.33 (0.91–1.94)	**1.38 (1.02–1.87)**	**1.49 (1.27–1.74)**	**1.25 (1.10–1.41)**	**1.69 (1.45–1.96)**	**1.72 (1.52–1.94)**
Age (years)						
15–24	1.00	1.00	1.00	1.00	1.00	1.00
25–34	1.08 (0.67–1.75)	1.24 (0.88–1.75)	**1.52 (1.23–1.87)**	**1.53 (1.32–1.79)**	**1.31 (1.08–1.59)**	1.38 (1.19–1.61)
≥ 35	0.83 (0.48–1.41)	0.75 (0.51–1.11)	0.93 (0.73–1.17)	**0.70 (0.59–0.83)**	**0.60 (0.48–0.75)**	**0.48 (0.40–0.57)**
Religion						
Buddhist	1.00	1.00	1.00	1.00	1.00	1.00
Muslim	0.38 (0.10–1.45)	0.73 (0.32–1.64)	0.87 (0.51–1.48)	0.91 (0.61–1.36)	**0.57 (0.34–0.95)**	0.75 (0.52–1.09)
Other	**0.09 (0.01–0.70)**	0.63 (0.06–1.23)	**0.42 (0.23–0.76)**	**0.72 (0.51–0.99)**	0.71 (0.45–1.12)	1.06 (0.79–1.44)
Residence						
Urban	1.00	1.00	1.00	1.00	1.00	1.00
Rural	1.16 (0.77–1.74)	0.92 (0.68–1.25)	0.97 (0.76–1.23)	0.96 (0.78–1.66)	**1.27 (1.01–1.59)**	**1.37 (1.13–1.66)**
Region						
Plains	1.00	1.00	1.00	1.00	1.00	1.00
Tonle Sap	**1.67 (1.13–2.47)**	**1.68 (1.25–2.24)**	**0.81 (0.66–0.99)**	0.98 (0.83–1.16)	1.14 (0.95–1.37)	1.15 (0.99–1.34)
Plateau / Mountain	**1.85 (1.17–2.93)**	**1.95 (1.39–2.74)**	**0.68 (0.54–0.86)**	0.90 (0.75–1.09)	0.99 (0.81–1.22)	1.06 (0.89–1.26)
Phnom Penh	**5.46 (2.71–10.99)**	**2.33 (1.27–4.29)**	1.18 (0.82–1.70)	0.99 (0.72–1.38)	**1.79 (1.29–2.49)**	1.06 (0.78–1.45)
Age at first marriage						
≤ 16	1.00	1.00	1.00	1.00	1.00	1.00
17–20	1.13 (0.77–1.67)	0.96 (0.73–1.25)	1.15 (0.96–1.38)	0.94 (0.83–1.06)	**1.21 (1.02–1.43)**	0.92 (0.81–1.04)
≥ 21	1.41 (0.92–2.17)	0.76 (0.55–1.04)	1.04 (0.86–1.27)	**0.76 (0.51–0.87)**	1.04 (0.86–1.24)	**0.67 (0.58–0.77)**
Total children ever born						
0	1.00	1.00	1.00	1.00	1.00	1.00
1–2	**6.39 (2.31–17.72)**	**8.90 (3.88–20.43)**	**5.57 (3.84–8.07)**	**18.21 (11.97–27.70)**	**5.67 (4.21–7.64)**	**20.28 (14.23–28.91)**
≥ 3	**8.92 (3.11–25.56)**	**10.31 (4.39–24.17)**	**6.52 (4.41–9.65)**	**21.53 (13.99–33.12)**	**8.25 (5.95–11.41)**	**28.92 (19.97–41.88)**
Wealth						
Poor	1.00	1.00	1.00	1.00	1.00	1.00
Middle	1.20 (0.80–1.80)	1.26 (0.94–1.69)	**1.33 (1.09–1.63)**	**1.15 (1.00–1.32)**	0.97 (0.80–1.18)	0.94 (0.82–1.09)
Rich	1.27 (0.80–2.01)	**1.42 (1.01–1.98)**	1.20 (0.97–1.49)	0.89 (0.76–1.04)	1.05 (0.85–1.29)	0.91 (0.77–1.06)
Media exposure						
No	1.00	1.00	1.00	1.00	1.00	1.00
Yes	1.32 (0.93–1.89)	1.22 (0.94–1.57)	1.02 (0.88–1.19)	1.06 (0.95–1.18)	1.00 (0.87–1.16)	0.98 (0.88–1.09)
Perceived distance to HF						
Big problem	1.00	1.00	1.00	1.00	1.00	1.00
No problem	**1.65 (1.21–2.25)**	1.09 (0.87–1.36)	**1.30 (1.12–1.50)**	1.09 (0.98–1.21)	**1.24 (1.08–1.42)**	1.08 (0.97–1.20)
Health insurance						
No	–	–	1.00	1.00	1.00	1.00
Yes	–	–	**0.76 (0.61–0.95)**	**0.85 (0.74–0.99)**	0.95 (0.79–1.14)	**1.23 (1.07–1.41)**
Partner education						
No formal education	1.00	1.00	1.00	1.00	1.00	1.00
Primary	0.88 (0.56–1.37)	1.19 (0.86–1.63)	**1.56 (1.21–2.02)**	**1.23 (1.06–1.43)**	1.26 (0.98–1.62)	1.04 (0.88–1.22)
Secondary and higher	1.30 (0.79–2.14)	1.28 (0.88–1.87)	**2.11 (1.61–2.76)**	**1.34 (1.13–1.58)**	**1.51 (1.16–1.97)**	1.17 (0.98–1.40)
**Community-level factors**						
Community wealth						
Low	1.00	1.00	1.00	1.00	1.00	1.00
Middle	0.83 (0.55–1.26)	0.99 (0.74–1.35)	**1.51 (1.20–1.91)**	1.00 (0.84–1.19)	1.11 (0.90–1.37)	1.04 (0.89–1.23)
High	0.99 (0.56–2.65)	0.92 (0.60–1.41)	**1.86 (1.35–2.58)**	1.01 (0.78–1.30)	1.35 (0.99–1.85)	1.22 (0.94–1.65)
Community education						
Low	1.00	1.00	1.00	1.00	1.00	1.00
Middle	1.41 (0.94–2.11)	1.25 (0.93–1.68)	**1.16 (0.92–1.46)**	0.94 (0.79–1.12)	**1.37 (1.12–1.68)**	0.91 (0.78–1.06)
High	1.05 (0.67–1.65)	0.98 (0.71–1.37)	1.12 (0.87–1.44)	0.84 (0.70–1.02)	**1.42 (1.14–1.76)**	0.86 (0.72–1.02)
Community decision-making						
Low	1.00	1.00	1.00	1.00	1.00	1.00
Middle	0.91 (0.61–1.37)	0.99 (0.74–1.35)	**0.76 (0.62–0.94)**	0.98 (0.83–1.16)	1.01 (0.84–1.22)	1.01 (0.87–1.19)
High	1.20 (0.80–1.80)	1.13 (0.83–1.53)	**0.65 (0.52–0.82)**	0.93 (0.78–1.11)	1.10 (0.91–1.34)	0.99 (0.84–1.16)
Community workforce participation						
Low	1.00	1.00	1.00	1.00	1.00	1.00
Middle	0.74 (0.50–1.10)	1.29 (0.97–1.73)	0.92 (0.74–1.14)	1.06 (0.90–1.26)	**1.31 (1.07–1.60)**	1.02 (0.87–1.19)
High	0.88 (0.58–1.32)	1.29 (0.95–1.76)	0.82 (0.65–1.04)	1.07 (0.89–1.29)	**1.41 (1.12–1.77)**	0.97 (0.81–1.18)
**Measures of variation**						
Area variance (95% CI)	0.55 (0.26–1.59)	**0.32 (0.15–0.67)**	**0.37 (0.27–0.51)**	**0.30 (0.24–0.39)**	**0.24 (0.17–0.36)**	**0.20 (0.15–0.28)**
ICC (%)	14.32	8.86	10.11	10.31	6.80	5.73
PCV (%)	51.33	33.33	51.95	10.71	51.02	5.26
MOR	2.03	1.72	1.79	1.70	1.60	1.53
**Model fit statistic**						
AIC	4258.33	4258.33	19,634.60	19,634.60	20,406.73	20,406.73

*RRR* Relative risk ratio*, CI* Confidence internal, *ICC* Intraclass correlation coefficient, *MOR* Median odds ratio, *PVC* Proportional change in variance, *AIC* Akaike information criterion

Having a high decision making power was associated with use of traditional contraceptive methods in 2010 [aOR: 1.71, 95% CI: 1.18–2.47] compared with those having low decision making power.

Across all survey waves, having high work participation was associated with increased odds of using modern contraceptive methods (Table 3). However, having medium workforce participation level was associated with use of both traditional methods and modern methods only in 2010 [aOR: 1.48, 95% CI: 1.25–1.76 and aOR: 1.24, 95% CI: 1.10–1.31, for traditional and modern methods, respectively] and 2014 [aOR: 1.53, 95% CI: 1.29–1.82 and aOR: 1.45, 95% CI: 1.27–1.65, for traditional and modern methods, respectively] surveys.

At the community level, women from communities with a middle [aOR: 1.37, 95% CI: 1.12–1.68] and high [aOR: 1.42, 95% CI: 1.14–1.76] percentage of educated women were more likely to use traditional methods compared to those from communities with a low percentage of educated women in 2014 survey. Meanwhile, in women from communities with a middle [aOR: 0.76, 95% CI: 0.62–0.94] and high [aOR: 0.65, 95% CI: 0.52–0.82] percentage of women having decision making powers were less likely to use traditional methods in 2010 survey.

#### Other factors associated with of contraceptive methods

Tables 2 and 3 further reveals other factors that were associated with use of any contraceptive methods, and specific contraceptive method, respectively.

Among others, across all survey waves, older age at first marriage was associated with reduced likelihood of using any contraceptive method while having > 1 child was associated with increased odds of using any contraceptive methods. In 2014, those that lived in rural areas were more likely to use any contraceptive methods. Regional variations were observed in 2005 in terms of use of any contraceptive methods while this variation was not significant in the other survey waves. Having a health insurance was associated with increased odds of using any contraceptive methods in 2014. Having a partner with primary education was associated with increased odds of using any contraceptive methods in 2010 (Table 2).

Women aged ≥35 years were less likely to use modern methods in 2010 while those aged 25–34 were more likely to use both traditional and modern methods compared with women aged < 25 years. On the other hand, in 2014, women aged ≥35 years were less likely to use both traditional and modern contraceptive methods compared with those aged < 25 years. Other factors that were associated with specific type of contraceptive method use included; religion, area of residence, region, age at first marriage, number of children ever born, distance to health facility, and having a health insurance.

#### Random effects

Measures of variation for the binary and 3-level outcomes are listed in Tables 2 and 3, respectively. The final models revealed significant variances for the outcomes in all survey waves. The MOR for all the survey waves for the binary outcome displayed the effects of community heterogeneity indicating that if a woman moved to a community with a high probability of using any contraceptives, the median increase in the odds of using any contraceptives would be 1.58 in 2004, while 1.66 in 2010, and 1.50 in 2014 (Table 2). Residual heterogeneity in the outcomes was observed as seen by significant variances in all the final models as well as the ICCs that displayed that there were still some unmeasured community factors, that could influence contraceptive use, that were not included in the current analysis.

## Discussion

This study examined the influence of women empowerment factors at both individual- and community- level on contraceptive use among married Cambodian women. Further, the study examined other relevant individual- and community- level factors that may be associated with contraceptive use among Cambodian women. Notably, educational attainment and participating in workforce were associated with increased likelihood of using any contraceptive method as well as specific contraceptive methods particularly in 2010 and 2014. The study also demonstrated that there are other unmeasured community factors that may influence contraceptive use among Cambodian married women.

It was observed that the use of modern methods increased over the survey waves from 2005 at 29.0 to 42.3% in 2014. FP programs in Cambodia have focused on improving awareness and knowledge, building capacity of midwives to provide contraceptive choices, increasing FP choices through community-based distribution of contraceptives, and enhancing a secure supply of commodities [39, 40]. Therefore, the increase in the use of modern methods may underline that the efforts and programs aimed at improving access to reproductive health care in Cambodia are making significant strides. However, more needs to be done as the reported 42.3% (2014) is relatively lower than the regional rate (68.0%) reported for Asia in 2015 [41]. As such, it may be prudent for Cambodia to learn lessons from other Asian countries that are making good progress with regards to contraceptive use.

Empowering women has been shown to influence their health behaviors in previous studies [42, 43]. In the current analysis, it was revealed that educated women and those involved in workforce were more likely to use contraceptive methods. Educated women are better informed about the various methods available for fertility control and may have greater geographical and financial access to contraception and overall reproductive health services [44]. Participating in workforce may empower women economically. Working women are more likely to have access to their own spending money hence they have a greater opportunity to use those funds towards family planning and reproductive health service utilization [45]. The results of these empowerment measures were similar even after examining their associations with specific types of contraceptive methods. Findings from the current research revealed the importance of women’s education attainment and workforce involvement in Cambodia with respect to the use of contraceptives. No significant findings were observed for decision making and contraceptive use albeit in 2010 where it was associated with the use of traditional contraceptive methods. Previous research in Cambodia identified women’s access to new knowledge and abilities, which technically helped them to be involved in income-generating activities, as a key empowerment aspect mentioned by Cambodian women [46]. This may partly explain why educational attainment and workforce involvement had greater influences on the use of contraceptives than decision making.

Our findings suggested that women from communities with a high percentage of women in workforce were more likely to use any contraceptive method. It was also observed that women from communities with a high percentage of educated women were less likely to use any contraceptive methods. The significant effect of community education disappeared when the multinomial models were run to examine the association with specific type of contraceptive method and community-level women’s decision making power then became negatively associated with contraceptive use. The results about the negative relationship between some community SES factors and contraceptive use is consistent to a Zimbabwean study [47], and a multi-country study that observed a negative association between educational attainment in community and contraceptive use [48]. They suggested that the level of education does not mediate the pro-natalist norms prevalent among women in local communities. Although unexpected, this finding may be partially explained by the fact that social and cultural norms may still play an important role in influencing contraceptive use within communities [49]. As such, women in communities where negative influence on contraceptive use exist may be discouraged to adopt the contraceptives. More research is needed to understand such relationships.

Several other individual-level factors were considered. Older women (> 34 years) were less likely to use contraceptives while those aged between 25 and 34 were more likely to use contraceptives. The findings are in line with results reported in Iran [50]. Older women’s awareness regarding their declining fertility could be one of the reasons why they are less likely to use both contraceptive methods. Consistent to previous research [51], age at first marriage was associated with contraceptive use with those aged ≥21 years at first marriage being less likely to use contraceptives. Women who married later in life may have the desire to bear children in the earliest time possible therefore, they may not prefer to use any contraceptive methods. Regional variations were observed in terms of contraceptive use. Additionally, in contrast to previous research [50, 51], women from rural areas were more likely to use contraceptives. In Cambodia, programs relating to reproductive health may be specifically promoted in the rural areas than urban areas and this may explain the findings in the current study. It was observed that in 2010, having health insurance was negatively associated with contraceptive use while in 2014 there was a positive association. Continuous improvements to health insurance system over time could be the reason for the observed differences between 2010 and 2014. Women that did not perceive distance to the nearest health facility as a problem were more likely to use contraceptive methods. This underscores the importance of improving health care services access as it is a precursor to accessing reliable information including those related to contraceptive use.

### Policy/program implications

First, strategies aiming at improving reproductive health in Cambodia should aim to integrate with efforts/programs that are geared to empower women as this may ultimately help improve utilization of contraceptives. Second, the study further revealed that other unmeasured community factors may influence contraceptive use among Cambodian women suggesting the need for public health programs to profile communities when designing or formulating their FP policies/programs. This may help in the development of tailored programs that may eventually be effective. Third, regional variations were observed in terms of contraceptive use suggesting that programs should focus on regions that are lagging behind. Fourth, improving contraceptive use in Cambodia requires a multifaceted approach with both the individual- and community- level factors identified in the current analysis being crucial to the implementation of effective programs.

### Strengths and limitations

The study used three survey waves with nationally representative samples which allowed for generalizability of the results to the wider population of Cambodian married women. Additionally, the findings on the association between empowerment variables and contraceptive use across the survey waves help to strengthen the relationships observed. The assessment of variables at different levels allowed the study to account for community differences as well as identify the existence of other unmeasured community factors which is important in the design of public health programs and future research, respectively. However, the study design precludes inferences of causality. There were other factors, as observed, that may account for variation in contraceptive use (such a community outreach, engagement, and mobilization efforts) that were not included in the survey.

## Conclusion

The factors influencing use of contraceptive methods (traditional or modern) among married Cambodian women operate at both individual and community level. Efforts to promote modern contraceptive use should aim at empowering women with a particular focus on improving access to education and employment opportunities. We recommend that through the avenues of education and increased awareness on personal rights, women would have a greater ability to negotiate with their husbands/partners to come to conclusions on contraceptive use that is best for both sides. Further, increasing labor market opportunities to give women economic independence. This could empower women and change social constructs for a more gender-equal world could have far-reaching positive effects that stem beyond improvements on reproductive health behaviors. To highlight the underlying cultural themes at work, as well as other unmeasured community factors, more research should be incorporated into future research for a more complete picture.

## Data Availability

The study used, with permission, data from the International Classification of Functioning, Disability, and Health (ICF). The data is publicly available upon request from the ICF on (https://dhsprogram.com/data/available-datasets.cfm).

## Abbreviations

FP: Family planning
CDHS: Cambodia demographic and health survey
EA: Enumeration area
aOR: Adjusted odds ratio
CI: Confidence interval
ICC: Intraclass correlation coefficient
PCV: Proportional change in variance
MOR: Median odds ratio
AIC: Akaike information criterion
DHS: Demographic and health survey
